# A systematic review of foraging as lifestyle, livelihood, and landscape management strategy

**DOI:** 10.1007/s13280-025-02222-9

**Published:** 2025-08-14

**Authors:** Mallika Sardeshpande, Tafadzwanashe Mabhaudhi

**Affiliations:** 1 Centre for Transformative Agriculture and Food Systems, School of Agriculture, Earth, and Environmental Sciences, University of KwaZulu-Natal, Scottsville, 3209 South Africa; 2 Ashoka Trust for Research in Ecology and the Environment, Bengaluru, India; 3 Centre on Climate Change and Planetary Health, London School of Hygiene and Tropical Medicine, London, UK

**Keywords:** Health, Human ecology, Social–ecological resilience, Sustainable development, Sustainable livelihoods, Wellbeing

## Abstract

This systematic review of 353 studies evaluates the knowledge on foraging by humans, situating it in the wider context of human ecology. We highlight the strengths and weaknesses, and the micro (individual) to macro (landscape) level implications of foraging, as concerns livelihoods and social–ecological systems. Descriptive statistics of ethnobotanical studies yielded 1410 genera foraged globally. Foraging can contribute to food and nutritional security, human health and wellbeing, adaptation to global environmental change, and good governance. Research priorities include establishing baselines for species suitability, nutritional quality and biophysical tolerance, social utility, innovation, and foraging impact. Policy recommendations include integrated spatial planning and supporting devolved local economies nested within larger governance and market frameworks to enhance human and natural capital and social cohesion. Actions to foster social–ecological resilience include improving access to forageable resources and spaces, sharing information on sustainable foraging, and landscape stewardship through sustainable foraging.

**Supplementary Information:**

The online version contains supplementary material available at 10.1007/s13280-025-02222-9.

## Introduction

Foraging is the act of an organism procuring a resource, usually food, necessary for its subsistence, from its environment. Commonly visible instances of foraging include livestock grazing in pastures (Philp et al. 2019), bees in fields and gardens (Lanner et al. 2020), and gulls in coastal areas (Marteinson and Verrault 2020). The heuristics of foraging have been studied in many motile organisms, from bacteria to humans (Serieys et al. 2019; Zapata et al. 2019, Found and St. Clair 2020). As humans have largely adopted relatively sedentary lifestyles and agrarian modes of production, foraging as a livelihood activity has been reportedly relegated to minor sections of society, such as forest-dwellers, nomadic hunter-gatherers, small agroecological farmers, and urban scavengers (Chohaney et al. 2016; N’Danikou et al. 2017; Paddeu 2019; Toffolo et al. 2019). Research on the phenomenon of foraging by humans has been ongoing in the fields of anthropology, economics, ethnography, natural resource management, and sociology (Reyes-Garcia et al. 2018; Morelli et al. 2019; Shackleton and de Vos 2022).

Foraged resources can help households reduce monetary expenditure on food, healthcare, and other materials (Wunder et al. 2014; Sanchez-Badini and Innes 2019), and the collection and sale of foraged resources can contribute to monetary income and savings (Poe et al. 2013; Shackleton et al. 2017a; Landor-Yamagata et al. 2018). Foraged resources may aid relief and recovery during social or ecological shocks, such as loss of livelihood, farm failure, or natural disaster (Erskine et al. 2015; Hofman 2016). Foraged foods are often fresher and nutritionally diverse than mass-produced and store-purchased foods (Ahmed et al. 2022, Nisbet et al. 2022). Thus, foraging can be an effective financial and nutritional safety net for many households worldwide. Humans across the social spectrum forage unembodied resources such as information and recreational experiences (McCullough 2013; Vaittinen and McGookin 2016). Foraging behaviour patterns in non-humans can be attributed to various intrinsic and extrinsic variables (Jacquier et al. 2020; Marty et al. 2020; Mimet et al. 2020). In contrast, human foraging is understood to be less heuristic and more relational. For example, for some people, foraging is a cultural and spiritual act connecting them to their origins and environment (Hurley et al. 2015; Elands et al. 2019; Nyman 2019). Thus, foraging may offer humans intangible rewards on effort invested, including mental wellbeing and social cohesion, which are difficult to economically quantify but are integral to existence.

Foraging occurs across various ecosystems, including wilderness and fallow areas, smallholder farms, and urban greenspace (Rupprecht et al. 2015; Shackleton and de Vos 2022). This presents the threat of overexploitation and opportunities for use-based conservation of resources in these ecosystems (Sardeshpande and Shackleton 2020). Foraging may provide alternative modes of production and consumption in a world where large-scale industrial agriculture is increasingly challenging due to climate change (Kremen and Merenlender 2018; Kummu et al. 2020). Knowledge of the economies of scale, diversity and sustainable use thresholds of foraged species, and the prevalence of practices across landscapes is fragmented across different research domains. This review seeks to better understand the current status of the knowledge on foraging by humans and establish the gaps, gluts, and research priorities. The a priori research questions are: (1) who forages? (2) what is the status of foraging ethnobiology and knowledge transmission? (3) why do humans forage? (4) where does foraging occur? and (5) how does foraging interface with landscape governance? An inductive thematic approach is used to code and synthesise the literature. Descriptive statistics of ethnobotanical and ethnobiological studies document species diversity, their parts, and their global uses in biomes and communities.

## Materials and methods

### Identification

**Fig. 1:**
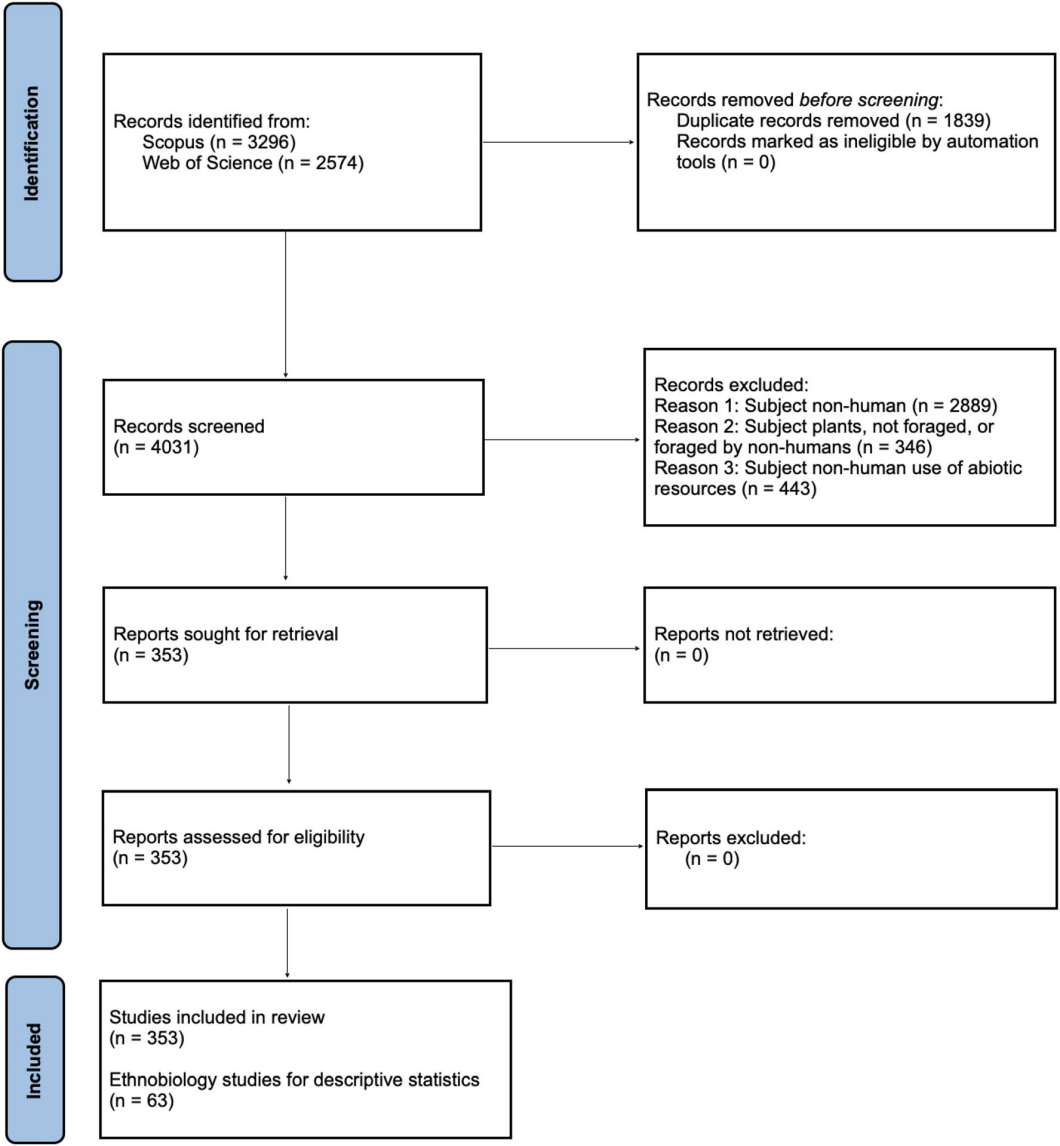
PRISMA flow diagram of protocol and criteria used to code, classify, screen, and shortlist articles for the review

The review used a PerSPEcTiF framework to formulate the search strategy and qualitatively evaluate the complex phenomenon of foraging. The PerSPEcTiF framework investigates phenomena from specific perspectives, within a given setting, environment, time frame, and comparison (Booth et al. 2019). In this review, the research question would be, from the perspective of human ecology, in a global setting, how does foraging across diverse environments compare with different forms of livelihood strategies and resource economies, for human health and wellbeing? The string ‘urban’ OR ‘peri-urban’ OR ‘rural’ AND ‘forage’ OR ‘foraging’ was used to retrieve literature for all time from the Scopus and Web of Science databases in June 2020. Articles containing these words in the title, abstract, and or keywords were included in the exported results (Appendix S1). The June 2020 retrieval was updated in November 2022 and April 2025 to include recent literature. The PRISMA protocol (Page et al. 2021) was used to conduct the review (Fig. 1), using articles in English only.

The string used to retrieve the literature was intended to capture foraging by humans across social–ecological gradients. We recognise that the formulation of the search string may have limited retrieval on two fronts. Firstly, articles that simply refer to foraging, without an environmental context (e.g. foraging around farms and forests): we posit that the rural–urban gradient enabled us to locate the literature on foraging by humans, as distinct from other animals, more efficiently; representativeness concerns were alleviated, given that two-thirds of the relevant studies were based in rural settings. Secondly, articles that use the terms ‘collect-’, ‘gather-’, or ‘hunt-’, which may be used to refer collectively to foraging: we argue that searching for these terms would require further qualifications of what is foraged (e.g. non-timber forest products, medicinal aromatic plants, firewood, etc.), and could potentially detract from the human ecology perspective (e.g. focus on resource biology, collection or hunting of non-wild resources). Acknowledging these limitations, we believe this review offers sufficiently contextualised insight into the phenomenon of foraging by humans as documented in the literature, and its significance to sustainability and resilience.

### Screening (exclusion, inclusion, and classification)

The inclusion criteria were narrowed down to studies with human subjects (not related to biological and nutritional human development or crop or livestock farming) and any clades with cultural, ethnobotanical, medicinal, nutritional, and socioeconomic value to humans (but not for farmed food, fuel, or livestock). Livestock, pastoralism, and crop science studies were deliberately excluded from the review due to the use of the term ‘forage’ being related to livestock systems rather than humans. Using these inclusion criteria, all articles spanning were manually scanned for articles relevant to human foraging in urban, peri-urban, and rural areas. The screening resulted in 353 (9% of the total) articles shortlisted for review from the retrievals (Appendix S1). Shortlisted articles were classified based on the ecosystems they were situated in (rural, urban, rural–urban gradient).

### Synthesis

**Fig. 2:**
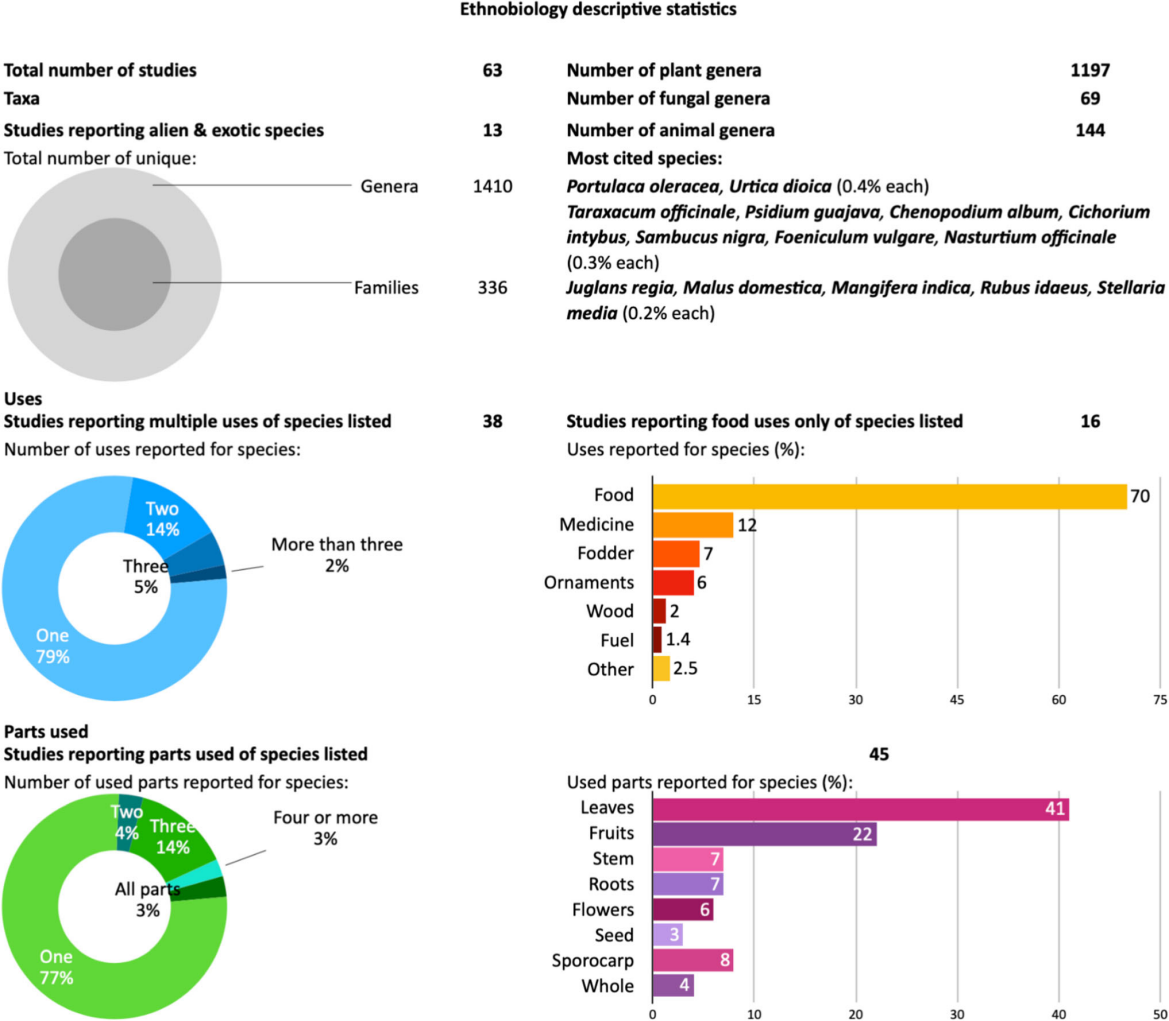
Results of the ethnobiology descriptive statistics (species list in Appendix S2)

The thematic analysis was framed using social–ecological systems theory (Cox et al. 2014), where resource users are foragers, resource units are foraged resources, resource systems are forager household economies, ecosystems are the foraging environment, and governance systems are the collective contexts within which foraging occurs (Table 1). Themes are not mutually exclusive. The 353 shortlisted articles were coded using ten emergent themes for resource users (foragers) and one theoretical framing each for the resource system (household economy) and ecosystem (foraging environment) (Table 1). Autecological and ethological examples are presented as a summary of the resource users (Table 2). Ethnobiological descriptive statistics summarise the resource units (Fig. 2, Appendix S2). The sustainable livelihoods framework (Serrat et al. 2017) was used to code the literature on socioeconomic drivers and implications of foraging (resource system), and the landscape ecology framing (Hersperger et al. 2021) was used to code the literature on environmental drivers and implications of foraging (ecosystem). The landscape management, institutional and policy aspects of foraging were combined into a hybrid section on the collective context of foraging (governance system). A further synthesis is presented using the sustainable livelihoods framework (Table 3), the sustainable development goals (Table 4), and social–ecological resilience actions (Table 5).

**Table 1:**
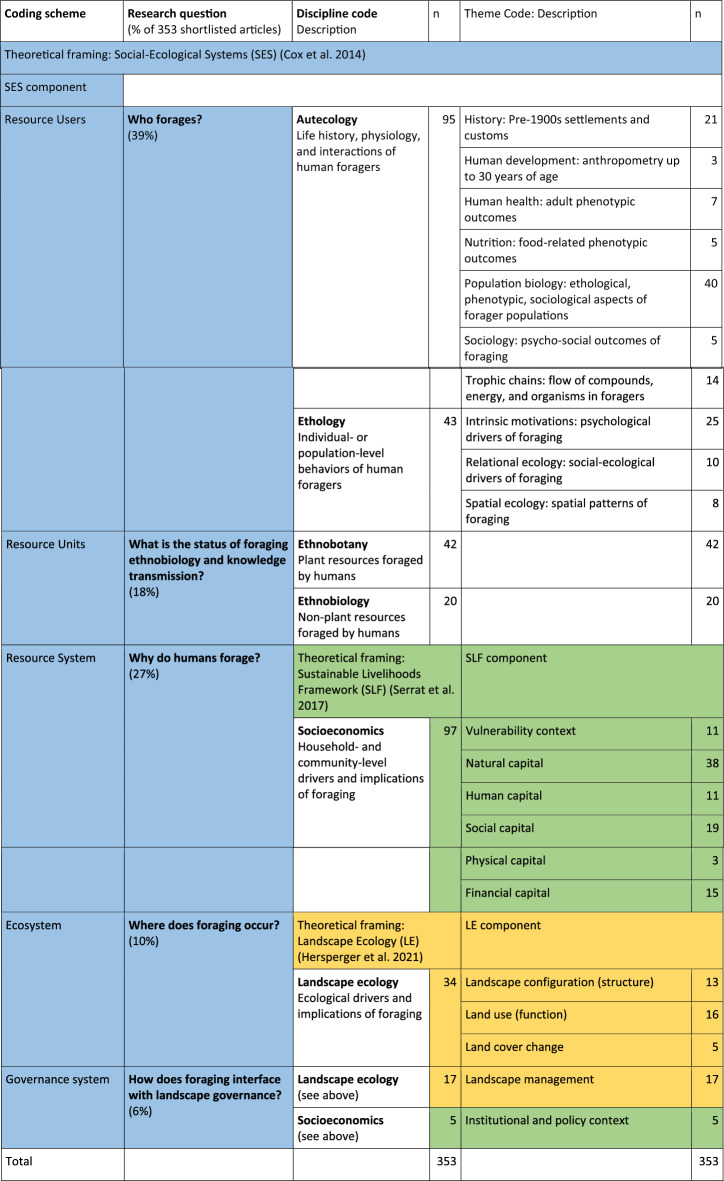
Distribution of articles across research questions, theoretical framings, discipline codes, and theme codes. Discipline codes are primary and mutually exclusive. Theme codes are necessarily secondary subsets of discipline codes. Tertiary sub-theme codes (indicated in Appendix S1) are independent of discipline and theme codes, and overlap across themes. Colours indicate components of theoretical framing aligned to research questions and themes

**Table 2:** Examples of recent and present-day human foragers, their locations, and motives

Foragers (*Tribes*) (examples from N = 277 articles)	Form and function (Main motive, frequency)	Other drivers and motives (Secondary reasons)	References (examples from N = 277 articles)
*!Kung* (Southern Africa); *Hadza* (Tanzania); *Ho (India); Mapuche, Toba/Qom* (Argentina); *Au, Anguganak, Bogasip, Wola* (Papua New Guinea); *Tawahka* (Honduras); *Shuar, Tsimané* (Bolivia); *Araweté, Kayapó, Asuriní* (Brazil); *Punan* (Indonesia); *Mikea* (Madagascar); *Baka, Efe* (Congo); *Coast Salish* (Canada)	Provisioning, primary lifestyle	Human (food, health), social (culture, recreation), physical (assets), financial (economic) capital, perception of spirituality, weather	Draper and Kranichfeld (1990); Tracer (1991); Jones et al. (1992); Ladio and Lozada (2001); Wong and Godoy (2003); Tracer (2004); Dounias et al. (2007); Godoy et al. (2009); Morsello and Ruiz-Mallén (2013); Tucker et al. (2015); Goetz and Valeggia (2017); Reyes-Garcia et al. (2018); Morelli et al. (2019); Toffolo et al. (2019); Urlacher et al. (2021); Kapoor et al. (2024)
*Viliui Sakha* (Russia); *Tekna* (Morocco); Grima (Central African Republic)	Seasonal nomadic agro-pastoral livelihood	Social capital, perception of access, weather	Crate (2008); Blanco et al. (2017); Schmitt et al. (2024)
Nineteenth century and present-day Blacks (USA); Karretjie Khoisan (South Africa); Indigenous Aborigines (Australia)	Historic injustice, subsistence and supplementation	Present-day expression of cultural heritage	De Jongh (2002); Clarke (2018); Lindemann (2023)
British Islanders (UK); Senior citizens (Albania, Croatia, Siberia); Farmer households (Timor-Leste)	Safety net during: World War II siege; mass migration; drought, and famine	Expression of economic and food sovereignty	Crate (2008); Erskine et al. (2015); Hofman (2016); Forster (2023)
Farmer households (Ghana; Kenya; Morocco)	Routine subsistence and supplementation	Expression of cultural heritage, knowledge, land stewardship	Boafo et al. (2014); Mganga et al. (2015); Genin et al. (2018)
Farmer households (China; Italy; Latvia)	Market diversification (food, medicine)	Market demand for: medicinal herbs; niche products	Buntaine et al. (2007); Grivins and Tisenkopfs (2018); Schunko et al. (2019)
Various rural communities (Global)	Routine subsistence and supplementation for food, fuel, and fibre near homes and forests	Perceptions of resource access, abundance, apparency, flavour	Shackleton et al. (2010); Hermans-Neumann et al. (2016); Soldati et al. (2017); Chaves et al. (2020)
Coastal communities (Puerto Rico); Small-scale fishers (Brazil); Women (Solomon Islands)	Artisanal fishing and coastal foraging for subsistence and supplementation	Human and social capital, economic and food sovereignty	García-Quijano et al. (2015); Keppeler et al. (2020); Bruckner and Paia (2024)
Various native, immigrant, and underprivileged urban citizens (Global)	Routine subsistence and supplementation for food, fuel, and fibre near homes and parks	Human and social capital, economic and food sovereignty	Chohaney et al. (2016); Synk et al. (2017); Martin (2018); Paddeu (2019); Fischer and Kowarik (2020); Lambino (2023); Ihle et al. (2024)

**Table 3:** A synthesis of studies comparing aspects of foraging and industrial resource production mapped on the sustainable livelihoods framework (Serrat 2017)

Sustainable livelihood criterion	Foraging		Industrial production		References from review results (N = 353)
	Pros	Cons	Pros	Cons	
*Vulnerability context*					
Shocks	Access to basic necessities and cash income; diverse sources increase resilience	Open access and sudden deficits reduce the reliability of resource availability	Accumulated stocks may alleviate the effects	Access to these stocks may be dictated by socioeconomic capacity and status	Shackleton et al. (2010); Erskine et al. (2015); Mann et al. (2020); Oncini et al. (2024)
Seasonality	Spatiotemporal and species diversification reduces the risk of systemic failure	Increased risk of variable returns on investment of effort	Reduced yield variability and lower risk of subsistence failure	Increased risk of location, species, or system-specific failures	Marston (2011); Lourme-Ruiz et al. (2022)
Critical trends	Adaptability in climate change and urbanising lifestyles	Scale mismatch between local and global economics	Optimising production and distribution efficiency	Large, unequal social and ecological impacts	Smith (2015); Clouse (2022); Lodge (2024)
*Capital assets*					
Natural capital	Valuing and propagating biodiversity through its use	Spatiotemporal variability may limit quantities of resources	Allows crops on an intensive scale, reduced sprawl of production	Inputs tend to be resource-intensive, and outputs pollute	Mganga et al. (2015); Synk et al. (2017); Naah (2020)
Human capital	Developing and propagating knowledge of resource use and ecology	This knowledge is informally passed on and increasingly lost to urban lifestyles	Knowledge advancement is geared towards industrial and technological improvements	Loss of primary biologically and culturally diverse technical know-how	Reyes-García et al. (2007); Kunwar et al. (2020); Mattalia et al. (2020); Sharman (2025)
Social capital	Shared rights, responsibilities, and resources enhance social cohesion, local economy, and human culture and wellbeing	Assimilation into capital and global economies may erode social capital	Improved energy and economic efficiency allow for social specialisation and complexity	Disconnect from sociology, processes, and landscapes of production creates social dissatisfaction and inequality	Burnside et al. (2012); García-Quijano et al. (2015); Tucker et al. (2015); Neto and Albuquerque (2018)
Physical capital	Communities that forage tend to share physical assets, including land, to fulfil needs	Industrial and privatised asset development inhibit shared ownership and governance	Development of strategic land and technological assets to aid efficiency	Disruption of traditional economic, tenure, and stewardship systems	Genin et al. (2018); Santika et al. (2019); Morrow and Martin (2019)
Financial capital	Cost and cash savings on resources, and income from sales	Resources may not always be acceptable or convertible to the cash economy	Relatively low-risk predictable and regulated returns on investment	Financial accumulation may acerbate socioeconomic inequality	Undurraga et al. (2016); Grivins and Tisenkopfs (2018); Ragie et al (2020)
*Institutional linkages*	Usually governed as commons	Governance structures are often implicit and not linked to institutions	Strong institutional structures and provisions can be mobilised	Power consolidation with institutions may marginalise alternative systems	Parry et al. (2009); Hofman (2016); Schunko and Brandner (2022); Sardeshpande and Shackleton (2025)
*Policies and processes*	Drivers, codes of conduct are embedded in local cultures and conditions	Globalisation and urbanisation threaten the loss of knowledge and adherence to local codes	Highly specific and wide-ranging policies and processes govern resource use	Scale mismatch between global trade and local conditions can cause scarcity and depletion	Rodríguez-Carreras et al. (2014); Larondelle and Strohbach (2016); Kowalski and Conway (2023)
*Livelihood outcomes*					
Food and nutritional security	Improved dietary and nutritional diversity, food security; richer body biomes and improved health	Socioeconomic and ecological constraints may restrict access to foraging spaces and species	Investment in a suite of species of high calorific and nutritional value, efficient value chains	Access to nutritious food dictated by affordability, knowledge, and availability	N’Danikou et al. (2017), Gupta et al. (2017), Garekae and Shackleton (2020), Bellows et al. (2023)
Health and wellbeing	Ability to practise traditional lifestyle and medicine; preventative, therapeutic benefits	Cultural, health, and wellbeing benefits are long-term and may not always be recognised by the mainstream economy	Increased stocks of calories and nutrients; efficient storage and distribution in the short-term	Adverse social, ecological, and cultural effects of production and supply chains; loss of knowledge	McLain et al. (2014); Goetz and Valeggia (2017); Sanchez-Badini and Innes (2019); Godoy et al. (2024); Ihle et al. (2024)
Sustainable resource use	Foraging is one component of various agro-pastoral and traditional ecosystem stewardship strategies	Market demand for some products may induce co-opting of species into unsustainable systems	Systemic links to technology, research, capital, and institutional and policy structures can be leveraged	High specificity and scale of systemic interventions reduce overall diversity and resilience	Buntaine et al. (2007); Blanco et al. (2017); Genin et al. (2018); Schunko et al. (2019); Davis et al. (2024); Aziz et al. (2024)

**Table 4:** The potential for diversity, resilience, sustainability, and conservation at different scales in the foraging social–ecological system (Cox 2014). SES, Social–ecological system; SDG, Sustainable development goal; CBD, Convention on biological diversity (Aichi target)

SES component	Diversity component	Resilience outcome	Recommendations	SDG	CBD
Resource users (Foragers)	Microbiome, lifestyle	Preventative lifestyle and immune benefits	Recognising and advocating diversity in food and livelihood systems: species, sources, and practices	1, 2, 3	1, 13
	Nutrition, diet	Food security, health, and wellbeing			1, 4
Resource units (Foraged resources)	Species	Ecological adaptation to shocks, change, scarcity	Propagating species and spaces of foraging value to improve user access and species abundance	15	7, 13
	Uses	User adaptation to shocks, change, scarcity		8, 10	1, 15
Resource systems (Foraging communities)	Economic systems	Savings on expenditure, social cohesion	Facilitating local economies and knowledge exchange through participatory research and governance	8, 10	4, 16
	Local, ecological knowledge	Sustainable use, cultural conservation		11, 12, 16	18, 19
Ecosystems (Commons)	Species	Overall biodiversity and functioning	Accounting for species and ecosystem service values in prioritising land use planning and policy	15	2, 13
	Services	Cultural, provisioning, regulating, supporting		11, 13	14, 16
Governance systems (Policies, structures)	Farms, markets	Reduced risk of failure, local resource efficiency	Valorising useful species for on-farm and market diversity and incentivizing sustainable stewardship	12, 16	7, 16
	Land use	Ecological stewardship, co-governance		15, 17	2, 5

**Table 5:** Suggested actions to conceptualise and operationalise social–ecological resilience guided by seven working principles (Sterk et al. 2017)

**#**	Resilience working principle	Social aspects	Ecological aspects	Related literature and examples from allied fields
		*Research priorities*	*Research priorities*	
		*Implementation priorities*	*Implementation priorities*	
1	Maintain redundancy and diversity (actors, resources, species)	Potential (innovation, collaboration) and limits (governance, markets) of systemic complexity	Scale and yields at varying degrees of land use complexity, sharing and sparing mosaics, and domestication and alien control potential	Wilhelm and Smith (2018); Ickowitz et al. (2019)
		Support localised and alternative governance and economies through education and inclusivity	Design and maintain locally appropriate mixed-use accessible spaces for ecosystem function	Säumel et al. (2016); Walsh-Dilley et al. (2016)
2	Manage social–ecological connectivity	Value and demand for species and spaces for foraging and related uses	Ecosystem tolerance to use, and opportunities for participatory restoration	Botzat et al. (2016); Gaoue et al. (2016)
		Create and facilitate the use of spaces and species for foraging through education and co-governance	Prioritise areas requiring greening, restoration, and wildlife corridors to complement land uses	Rupprecht et al. (2015); Menon et al. (2019)
3	Manage slow variables and feedback	Human (health and wellbeing) and social (cohesion, culture) capital under different governance regimes	Natural (biophysical, biodiversity) and physical (land use, ecosystem services) capital across regimes	Buijs et al. (2019); Goodwin (2019)
		Enrich food environments with diverse species and residential areas with multi-use spaces through market and planning instruments, respectively	Provide training and education on sustainable extraction practices based on conservative estimates and monitor ecological indicators over time	Kowarik (2018); Novello et al. (2018); Downs et al. (2020)
4	Foster complex adaptive systems thinking	Risk reduction, robustness, and adaptive capacity of localised socioeconomic systems	The adaptive capacity of foraging species and spaces under climate change scenarios	Augstburger et al. (2019); Cole et al. (2019)
		Policy provisions for nesting into larger economies of scale and governance institutions for support	Integrate foraging species and spaces with other types of green, adaptive, and built infrastructure	Artmann et al. (2019); Hansen et al. (2019)
5	Encourage learning and skill exchange	Local knowledge of species and sustainable use	Experimentally observed ecological response to use	Thomas et al. (2017)
		Document and disseminate social and ecological information on sustainable foraging through dialogue, education, and experiential learning		Sténs et al. (2019)
6	Broaden participation by active stakeholder engagement	Interdisciplinary dialogue in food & nutrition, health & wellbeing, sustainable livelihoods, agriculture, ecology, built environment, anthropology		Cocks et al. (2016); Bergius and Buseth (2019); Elands et al. (2019)
		Participatory planning and prioritisation of land use combinations to promote social cohesion, ecological stewardship, and sustainable governance with production efficiency and sufficiency		
7	Promote polycentric governance systems	Experiment with and apply different locally appropriate degrees of access and codes of conduct to govern foraging in public and communal infrastructure to promote multifunctionality and stewardship		Mantyka-Pringle et al. (2017); Winter et al. (2017)

## Results

### Who forages?

The first relevant article among the shortlisted 353 was published in 1981. The overall results did not contain reviews of the concept of foraging as a phenomenon, but contained one review specifically human foraging in urban areas in the Global North (Oncini et al. 2024). Research on human foraging is relatively emergent, recent, and sparse. Over half of the relevant literature was reported from rural settings (*n* = 204), just over a quarter (*n* = 99) was located in urban ecosystems, and about 14% (*n* = 50) was situated across the rural–urban gradient. The literature on resource users, focussing on human behaviour, ecology, and sociology, was the dominant theme, followed by socioeconomic drivers and implications of foraging (Table 1).

#### Autecology of human foragers

Table 2 summarises the specific examples and extents of foraging as a livelihood strategy for people worldwide. These examples range from tribes and societies that undertake foraging as a primary lifestyle to provision resources for their households and to share with their communities (Reyes-Garcia et al. 2018) to cosmopolitan natives and immigrants who forage as a recreational activity to preserve and perpetuate their culture (Palliwoda et al. 2017) and include sections of society that are spatiotemporally deprived of access to resources (Clarke 2018; Paddeu 2019).

Optimal foraging theory posits that organisms seek to maximise their foraged resources while minimising their energy expenditure (Dwyer and Minnegal 1985; Soldati et al. 2017; Páez et al. 2018). This theory has been used to explain the transition of human societies to agriculture as a primary livelihood to optimise food yields (Neto and Albuquerque 2018). For example, some foraging societies are believed to have facilitated the propagation of useful species, especially food-bearing plants, in the proximity of their dwellings, thereby reducing the need to travel in search of them (Fedick 1995). Cultivation of useful plants, and the eventual large-scale shift to agriculture are framed as a means of reducing the effort, risk, and uncertainty involved in foraging for these plant resources (Marston 2011). Further developments in the optimal foraging theory include the diet breadth model and the niche construction theory (Neto and Albuquerque 2018). According to the diet breadth model, agriculture gained momentum in response to increasing needs for energy and resources, and active interventions by humans in the social–ecological environment to meet them. On the other hand, niche construction theory proposes that agriculture spread due to the increased availability of energy and resources in the environment, allowing humans to develop knowledge and technology to improve outputs.

Both these theories attribute the uptake of agriculture to changes in human social organisation and its interventions. In contrast, the diet breadth model views the transition as an economical response to increasing population pressures, niche construction theory views it as a response to a surplus supply of resources (Burnside et al. 2012). The consensus is that foraging is a low-risk, low-returns, reflexive provisioning strategy from a pre-agrarian lifestyle (Smith 2015). Agroforestry and agroecology are forms of complementary farming and foraging that continue to exist today, albeit on a smaller scale, compared to industrial agriculture, on which many developed and urban societies rely.

Human communities optimise spatiotemporal resource extraction in industrial societies that, unlike most animals, utilise several times their metabolic capacity worth of energy (Burnside et al. 2012). Historically, humans used small-scale strategies to ensure the immediate availability of resources (through foraging) at the individual or household level. Complex urban networks redistribute control and consequences of resource extraction, removing individual or household responsibility to sustainably steward resources in a monetary economy (Smith 2015). Humans profoundly alter the ecology in urban areas through physical and cultural drivers, such that although the diversity and access of resources are higher in cities (compared to hunting and gathering in natural areas), an environment of easy access, especially to calorie-dense nutrient-poor foods, may result in malnourishment, weaker knowledge of food and acquisition skills, and lower body activity (Downey 2016; Goetz and Valeggia 2017; Tracer and Wyckoff 2020; Mateos et al. 2022). This, in turn, can lead to health and wellbeing deficits, further exacerbated by psycho-social pressures.

#### Intrinsic and relational motivations and patterns of foraging

Various intrinsic motives, such as personal and family values for culture and recreation, may influence foraging. Here, we report from the literature on individual and population-level motivations for foraging as an activity or lifestyle. This includes intrinsic and relational values and patterns that emerge from these choices. Economic motivations and implications of foraging at the household and community level are discussed in Sect. “Why do humans forage?”. Foraging as a behaviour may be deep-seated: for example, farmer-foragers across biomes and terrains (Reynolds et al. 2018) and visitors at dance and sports events (Rutten et al. 2021) move in remarkably similar patterns to foraging bacteria and other animals. The literature indicates that a primarily foraging lifestyle manifests in physiological differences from predominantly agrarian or urban lifestyles. This includes greater nutritional adequacy and diversity, physical fitness, and a richer, more diverse microbiome (Schnorr et al. 2014; Jha et al. 2018; Rowan et al. 2021). The diversity and richness of the forager microbiome renders them less susceptible to immune stress, inflammation factors, allergies, and lifestyle diseases (Turroni et al. 2016; Gupta et al. 2017; Klein et al. 2018).

Predominantly foraging societies tend to adopt and adhere to this lifestyle by choice, valuing it for cultural, social, and spiritual reasons (Table 2). For example, in the Anguganak and Bogasip of Papua New Guinea (Tracer 2004), the exchange of resources and services in the traditional economy is more prevalent and acceptable than monetary transactions. The Tsimane people of Bolivia regard monetary inequality more negatively than inequality in traditional assets (Godoy et al. 2024). Traditional knowledge and lifestyles are as important as formal schooling in some forager communities (Reyes-García et al. 2007; Morsello and Ruiz-Mallén 2013), indicating high regard for cultural heritage. Low integration into mainstream monetary economies can result in drastic inequalities for forager societies. For example, in coastal Madagascar, Mikea farmer-foragers were exploited for labour and produce in the market economy by their neighbouring farmer and fisher communities, resulting in significant income, social, and political inequality (Tucker et al. 2015). Income inequality may influence health and wellbeing and increase illness among marginalised communities, of which foragers may be part (Undurraga et al. 2010 and 2016, Tanner et al. 2013, Knight et al. 2021). These risks and inefficiencies notwithstanding, foragers continue to adhere to their lifestyles due to intrinsic motivations.

Many foragers report a sense of stability, place, community, and autonomy as integral to their lifestyle, identity, or life satisfaction (García-Quijano et al. 2015, Table 2). For example, immigrants from rural areas find that foraging helps them keep their cultural identity and connection to nature alive (McLain et al. 2014; Chou 2018; Martin 2018). The importance of foraging as a personal and social activity is also reported among non-forager communities who engage in some form of foraging, such as sport hunters, fisherfolk, and collectors, and even agriculturists and urban dwellers (Iveson et al. 2019; Chaves et al. 2020; Keppeler et al. 2020; Brouwer 2023; Lindemann 2023; Ihle et al. 2024). The recognition of common-pool resource rights and food sovereignty is a source of cultural, spiritual, and political fulfilment for some foragers (Hurley et al. 2015; Chou 2018; Iveson et al. 2019; Nyman 2019). Foraging can also provide preventative and therapeutic health benefits, especially among marginalised groups such as the disabled and disadvantaged (Wolf and Robbins 2015; Sanchez-Badini and Innes 2019; Ware 2022).

### What is the status of foraging ethnobiology and knowledge transmission?

The descriptive statistics of ethnobiology studies listing used species yielded over 2400 species belonging to 1410 unique genera, of which 85% were plants, 10% were animals, and 5% were fungi (Appendix S2, Fig. 2). A fifth of these species had more than one use, and a fifth had more than one useful part. A significant proportion of foraged species are native and of least concern, although there are examples of some exotic and endangered species being harvested, particularly for food (Landor-Yamagata et al. 2018, Fig. 2). The ethnobiological lists were sourced from 63 studies, documenting the plant (*n* = 43) and non-plant (*n* = 20) biodiversity foraged by humans. The studies are from over 25 countries, and distributed across 23 biomes. These studies documented local names in a total of 25 languages besides English and Latin. About a quarter of these studies (*n* = 16) were in urban landscapes, and very few (*n* = 6) were situated along the rural–urban gradient, with most (*n* = 41) being in rural areas. A majority of the studies were from Europe (*n* = 19), followed by South America (*n* = 13), Asia (*n* = 11), and Africa (*n* = 10), and North America (*n* = 10).

Knowledge of wild species is crucial for their nutritional and economic benefits to be utilised (Mattalia et al. 2020; Grivins 2021). For example, impoverished agrarian communities in biodiverse regions, malnourished urban poor, and farmers with urbanising lifestyles (Downey 2016, Fontefranceso et al. 2022) may be unable to forage resources due to lack of knowledge. Ethnobotanical knowledge is linked to improved household nutrition (McDade et al. 2007) and economic status (Godoy et al. 2005; Zhyla et al. 2018). Knowledge of species and spaces may, at times, manifest in responsible and sustainable foraging practices: some foragers tend to harvest the most abundant species (Soldati et al. 2017; Charnley et al. 2018; da Silva Santos et al. 2019; Fischer and Kowarik 2020), and in some cases invasive species, with an ethos of landscape stewardship (McLain et al. 2017; Landor-Yamagata et al. 2018; Ngorima and Shackleton 2019; Chaves et al. 2020).

Traditionally, knowledge about useful species is shared by elders with children and by children with their peers (McDade et al. 2007; Łuczaj and Kujawska 2012; Rosetti et al. 2016; Morelli et al. 2019). Frequent interaction with the natural environment is also a key conduit to ethnobiological knowledge (McNamara and Wertz 2021; Mlambo and Maphosa 2022). Other more recent means of knowledge transmission include the portrayal of foraging in the media (Sachdeva et al. 2018), outreach efforts by researchers (Stenchly et al. 2019), and print and social media (Sachdeva et al. 2018; Svanberg and Lindh 2019). Mobile and internet applications to store and disseminate information about foraging in urban areas, including locations, yields, and sustainability recommendations, are gaining popularity (Chamberlain and Griffiths 2013; Arrington et al. 2017; Disalvo and Jenkins 2017). Ethnobotanical studies and foragers and botanists are valuable repositories of knowledge on foraged species and spaces (Łuczaj and Kujawska 2012). Social foraging in urban green spaces is also a proliferating channel for knowledge transmission (McLain et al. 2017; Palliwoda et al. 2017; Riolo 2019). Planting biodiverse edible gardens and foraging walks on school campuses can facilitate learning about foraging, nutrition, and biodiversity (Fischer et al. 2019; Itchuaqiyaq and Matheson 2021).

### Why do humans forage?

Beyond intrinsic and relational motivations, foraging may be driven by the desire to cultivate human and social capital through education, local economy, and social cohesion, as well as accumulating physical or financial capital through savings or sales from foraged resources. Foraging allows several communities across the globe free access to resources such as food, fuel, and fibre for subsistence and livelihoods (Shackleton and de Vos 2022). These resources feed into household economies through environmental income or subsidies from nature, often saving people significant monetary expenditure on basic necessities (Wunder et al. 2014). Among primarily foraging communities, foraged resources contribute between 85 and 50% of the total household resource and cash income (Wong and Godoy 2003; Godoy et al. 2004). Even in agrarian communities, over a quarter of the annual household income may be from selling foraged plants and hunted animals (Boafo et al. 2014). In another example, a study from Japan finds that over time and across the rural–urban gradient, foraged food forms between 11% (rural) and 3% (urban) of household food economies (Kamiyama et al. 2023). Foraging and hunting may contribute directly and significantly to child nutrition (McDade et al. 2007), food security (N’Danikou et al. 2017), and dietary diversity (Garekae and Shackleton 2020) regularly in agrarian and urban communities. For example, a study found that across the UK, about a fifth of households that grow food also forage for it, and these households tend to consume significantly more fruits and vegetables than the average intake (Gulyas and Edmondson 2024).

Foraging protects the household economy against socioeconomic and ecological variation, scarcity, and shocks (Godoy et al. 2007; Erskine et al. 2015). In some cases, market demand can spur positive outcomes for conserving and monetising foraged agrobiodiversity while diversifying household income sources (Grivins and Tisenkopfs 2018; Schunko et al. 2019). Further, the unintended, natural, and multidimensional qualities of foraged resources can also translate into material value in urban contexts, taking the form of marketed goods and educational experiences (McLain et al. 2017; Iveson et al. 2019; Nyman 2019). Foraging provides communities with unique opportunities to engage in intergenerational knowledge transfer and interactive experiences in food production and biodiversity conservation (Poe et al. 2014; Palliwoda et al. 2017; Fischer et al. 2019; Riolo 2019; Fischer and Kowarik 2020). Foraging allows communities to share labour and resources to collectively manage their environment and produce goods and services (Bates 2013; Iveson et al. 2019; Morrow and Martin 2019).

Print and social media have transformed the public perception of foraging as a survival strategy for the poor and primitive to a leisure and luxury activity for the urban populace (Sachdeva et al. 2018; Svanberg and Lindh 2019). Thus, foraging has come full circle from supporting nomadic pre-agrarian populations to mobile non-agrarian urbanites, serving various tangible and intangible purposes at the interface of humans and their environment. Since the 1800s, human procurement and production of goods and services has attained industrial proportions, often creating abundance in certain geographies and deficits or degradation in social–ecological systems and subsequently livelihoods, elsewhere (Krausman and Lanthaler 2019, Rosa et al. 2023, Bruckner et al. 2023, 2024). Exploring the role of foraging as a livelihood, Table 3 presents the benefits and drawbacks of foraging against the sustainable livelihoods framework to contextualise economies of scale at household, community, and global levels. Table 3 extrapolates foraging and industrial production to all resources in general, but foraging and industrial food production are prime examples illustrating these pros and cons, with related literature from the results and the wider context cited in the references column.

### Where does foraging occur?

Structure, function, and change in a landscape influence foraging within the landscape. Seasonal adaptation is the primary ecological driver of foraging (Tables 2 and 3), followed by access to resources in the form of their ecological abundance and apparency (Hermans-Neumann et al. 2016; Soldati et al. 2017; Cordero 2025). Foraging is often undertaken in response to seasonal changes during which other livelihoods become less viable (Blanco et al. 2017) or when resources become more readily available (Genin et al. 2018). Ecological shocks like droughts and floods may also induce foraging (Erskine et al. 2015; Leakey 2018). Both in the short and long term, the visibility and accessibility of resources in an ecosystem positively influence foraging behaviour, with the more apparent and abundant species being foraged, be it in wild or urban spaces (Charnley et al. 2018; da Silva Santos et al. 2019; Wood et al. 2021). The species abundance and diversity of some landscapes are often a result of their spatial and structural configuration, such as being located in relatively less fertile, and undulating areas which are sub-optimal for conversion to large-scale farming or urban use (Nauhuelhuel et al. 2018; Starke et al. 2021; Garekae et al. 2022; Davis et al. 2024). As a result, various communities actively protect and propagate foraged species in their ecosystems to adapt to seasonal changes or shocks (Mganga et al. 2015; Makhubele et al. 2022). In some cases, this forms virtuous cycles wherein species diversity adds functional diversity to ecosystems, allowing agroforesters, agropastoralists, and even urban residents to steward productive, multi-purpose, adaptive landscapes (Nkem et al. 2013; Boafo et al. 2014; Hurley and Emery 2018).

Large-scale drivers of foraging include land use change related to industrialisation and urbanisation. Fertiliser and pesticide runoff and soil erosion from farms may affect the abundance or quality of foraged species or ecosystems (Mganga et al. 2015; Blanco et al. 2017; Amato-Lourenco et al. 2020; Chamberlain et al. 2024; Hanley et al. 2024). Foraging spaces may be converted to ‘more productive’ farmland, depriving communities of opportunities to forage or hunt and subsequently compromising their socioeconomic welfare (Parry et al. 2009; Santika et al. 2019). National or regional economic policy and changes may also result in significant landscape changes, promoting or constraining foraging (Rodríguez-Carreras et al. 2014; Rane and Ghule 2025). For example, government-sanctioned development of residential areas and roads may influence access to wild or formal greenspace in rural and urban contexts (Larondelle and Strohbach 2016; Clarke 2018). Migration of people away from rural and agrarian areas or shrinking urban areas can induce people to forage in abandoned land (Vullnetari and King 2008; Crate 2010; Hofman 2016; Hirahara 2020). The migration of people into urban areas is giving rise to a pronounced upswing in urban foraging (Shackleton et al. 2017a; Sardeshpande et al. 2021). Empirical evidence of the ecological implications of urban foraging is limited (Schunko et al. 2021; Davis et al. 2024; Prangel et al. 2024).

Urbanisation has often transformed erstwhile commons into private or public urban uses such as large-scale waste dumping and waterways diversions (Elkind 2006). Further, development planning or the lack thereof has reinforced the historic socioeconomic marginalisation of communities, often relegating marginalised groups to areas with limited private and common spaces and subsequently diminished opportunities to farm or forage to support their household economies (Poe et al. 2013; Sultana et al. 2020; Lindemann 2023). In some cases, the in-migration of wealthy urbanites leads to gentrification, formalising neighbourhoods, and discouraging or even outlawing foraging activity therein (McLain et al. 2014; Hurley et al. 2015; Charnley et al. 2018; Rane and Ghule 2025).

### How does foraging interface with landscape governance?

Regarding resource governance, foraging has long been recognised as a socially and economically important activity in rural livelihoods, and has been endorsed by various government schemes to rehabilitate landscapes and regulate ecosystem services (Raes et al. 2014; Hirahara 2020). However, such endorsement in the form of land use policy and incentivisation programmes needs to consider local needs and preferences for certain forms of agriculture, resource use, and integration into cash economies (Vermeulen et al. 2011; Curry 2019; Ragie et al. 2020; Shackleton et al. 2024). Participatory co-development of land use regimes is key to adopting livelihoods and sustainable resource use (Stenchly et al. 2019; Schossler et al. 2021; Reiss et al. 2024). In urban areas, foraging is usually not specifically governed or promoted by policies, but is subject to several other land use policies, especially concerning private property and greenspace management (Shortly and Kepe 2021; Garekae and Shackleton 2021, 2022; Kowalski and Conway 2023). These include planting protocol avoidance of fruiting species along roads and fences to minimise biomass hazards and clearing, using chemical herbicides, and air and water pollution in some places (McLain et al. 2014; Marquina et al. 2022; Sardeshpande and Shackleton 2025).

Nevertheless, there is growing recognition of the value of foraging in various spaces, ranging from urban forests and parks to sidewalks and vacant lots (Shackleton et al. 2017b). Some studies also identify concerns and commonalities between the goals and practices of formal urban greenspace management and foraging (McLain et al. 2017; Sardeshpande and Shackleton 2020, 2025). The emerging literature points to foraging as a possible nature-based solution to make cities more food secure, resilient to climate change, and liveable (Sardeshpande et al. 2021; Shackleton et al. 2024).

Foraging introduces or perpetuates social, ecological, and economic diversity, fostering systems resilience (Table 4). In common-pool resource theory framing (Ostrom 2009), the social–ecological system (SES) of foraging comprises foragers (resource users), foraged resources (resource units), communities where foraging occurs (resource systems), landscapes where foraging occurs (ecosystems), and the policies, processes, and structures which foraging is subject to (governance systems) (Cox 2014). The theory is a helpful framework for assessing the governance of common-pool resources and the SES in which they occur, with a view to sustainability (Fleischman et al. 2014). Foraged species and the spaces they occur in may also harbour wildlife in both rural areas (Parry et al. 2009; Tanner et al. 2013; Boafo et al. 2014; Reyes-García et al. 2018; da Silva Santos et al. 2019; Chaves et al. 2020) and urban settings (Poe et al. 2014; Bunge et al. 2019; Fischer et al. 2019; Riolo 2019; Sardeshpande and Shackleton 2020). Thus, conserving these species and spaces and promoting sustainable foraging could serve the dual purpose of provisioning and supporting human and non-human biodiversity. Foraging in urban areas highlights environmental justice and needs and can build on social capital and nested governance to empower citizens to co-produce their commons (Ballamingie et al. 2019; Morrow and Martin 2019; Riolo 2019; Sultana et al. 2020, Reiss et al. 2024). Therefore, we also make recommendations to the potential of foraging in contributing to sustainable development goals (SDG 2021) and the Aichi targets for biodiversity conservation (CBD 2021).

## Discussion

Our review finds that foraging by humans occurs across the rural–urban gradient and socioeconomic spectrum. It can be a primary or supplementary livelihood and a purely cultural and recreational activity. Thus, motives for foraging may include its contributions to the household economy through earnings and savings or its intangible value for health and wellbeing. Humans who rely significantly on foraged food tend to accrue dietary, nutritional, and fitness benefits. The main enablers of foraging are knowledge of foraging species, and access to foraging spaces. The main barriers to foraging are industrialised and urbanised landscapes. Gaps in the knowledge on foraging include the scalability and embedding of local foraging economies into mainstream production economies, and the ecological implications of foraging. Foraging is predominantly a food procuring activity (Fig. 2), and can provide multiple benefits for food and nutritional security, healthy food environments, and sustainable food systems.

It is acknowledged that foraging as a livelihood activity may in some cases co-occur with household poverty and vulnerability (e.g. Curry 2019; Mann et al. 2020, Nisbet et al. 2022). When compared to agriculture and market purchases, foraging is a relatively time and labour-intensive activity, which some households may resort to out of necessity rather than choice (Chakrabarti et al. 2023; Oncini et al. 2024). Nevertheless, much of the evidence indicates that foraging is usually a safety net, a step out of poverty, and a conscious choice for those engaging in it (Undurraga et al. 2016; Reyes-García et al. 2018; Grivins 2021; Sardeshpande and Shackleton 2023; Shackleton et al. 2024). The general literature contains examples of natural resources being extirpated by market demand precipitating overharvesting and poor practices (e.g. Hopping et al. 2018; de Alcantara et al. 2020). This review did not find such examples in the shortlisted articles. Where attempts have been made to estimate the ecological impacts of foraging, they are from a human perspective, indicating that foraging is undertaken with consideration for the continued vitality of the resource and the health of the landscape it occurs in, in both rural (Blanco et al. 2017; Makhubele et al. 2022) and urban (Brandner and Schunko 2022; Cordero 2025) contexts. Therefore, we reiterate the need for more research on the ecological impacts of foraging by humans across the rural–urban gradient with a social–ecological systems approach (Sardeshpande et al. 2021; Prangel et al. 2024; Sardeshpande and Shackleton 2025).

Knowledge of foraging species and spaces and their ecological interactions is critical and situation-specific. Foragers tend to use their knowledge to forage in time, space, and volume such that the ecosystems they interface with are minimally disrupted (Mantyka-Pringle et al. 2017; Thomas et al. 2017; Wynberg 2017; Novello et al. 2018; Guachamin-Rosero et al. 2022). Such knowledge should be developed and promoted with due respect to cultural considerations (Wynberg and van Niekerk 2014; Willcox et al. 2015). Where information does not exist, such as the yields, harvest tolerance, and value to wildlife of certain species or spaces of foraging interest, research needs to estimate and suitably quantify sustainable thresholds, for which empirical guidelines do exist (Venter and Witkowski 2013; Gaoue et al. 2016; Bunge et al. 2019). Our species list with biome and use specifications is a broad starting point for prospective foragers, land managers, and researchers.

For communities, foraging could provide equitable access to nutritious food, alternative medicine, biodiverse green spaces, and cultural and social capital (Kabisch et al. 2015; Kowarik 2018; Sanchez-Badini and Innes 2019). For health, food, and ecological systems, alternative pathways towards human development could be mainstreamed (e.g. domestication of useful species) or could reduce pressure on conventional systems (Russo et al. 2025). Improved access to green spaces enriched with forageable food species, and localised foraging gardens, will likely improve nutrition, health, and wellbeing, especially among deprived and vulnerable communities (Sardeshpande et al. 2021). Encouraging ecologically sustainable and co-managed foraging can reduce socioeconomic, structural, and systemic inequalities and improve access and benefit-sharing from ecosystems. Ripple effects of sustainable foraging are likely to aid the adaptive resilience of social–ecological systems by creating synergies across social, ecological, economic, and governance priorities (Handte-Reinecker and Sardeshpande 2025). The complexity introduced by such diversity and redundancy is likely to have limits, and research is required on pathways to nest localised economies into mainstream institutional frameworks (Table 5).

Foraging is often framed as an adaptive strategy to balance ecosystem variations over seasons or unforeseen events and diversify and localise the provisioning portfolio (Table 2). These livelihood features are increasingly recognised as important to systemic resilience, especially in response to climate change (Walsh-Dilly et al. 2016; Augstburger et al. 2019). Foraged species often grow with very little care and input. They are tolerant to climatic extremes and extraction (Scoles and Gribel 2015, Varghese et al. 2015, Lankoande et al. 2017, Mabhaudhi et al. 2017), making them attractive alternatives for cultivation either in conventional agriculture or alternative food spaces. Foraging embodies principles of agroecology, sustainable intensification, and devolved food networks, which are crucial transitions towards sustainable food systems (El Bilali et al. 2019; Russo et al. 2025). The emerging literature on edible urban landscapes and urban food production tends to focus almost entirely on agriculture or gardening using conventional crops (Boukharta et al. 2024; Yang et al. 2025), not taking into account the wild or underutilised species and alternative food economies associated with foraging. Even the literature on rural agrobiodiversity and agrarian economies does not specifically refer to these species or the practice of foraging as a conduit for seed saving, land stewardship, alternative livelihoods, or sustaining food networks (Hammond et al. 2023; Suomalainen et al. 2023; Wu et al. 2024; Wu and Zhang 2025). Foraging is a good example of integration of knowledge and praxis across different domains, which is essential towards improving land management, sustainable production systems, and planetary health (Fischer et al. 2025; Handte-Reinecker and Sardeshpande 2025; Russo et al. 2025). 

Foraging integrates multiple landscape components to form a mosaic provisioning system as opposed to a uniform production system, rendering it more resilient to shocks such as droughts, floods, fires, or political upheavals (Mbow et al. 2014; Hodbod and Eakin 2015; Balama et al. 2016; Larondelle and Strohbach 2016; Leakey 2018). Foraging is the middle ground in the land-sparing/sharing dichotomy as a livelihood strategy, offering scalable alternatives to extensive monoculture (Isbell et al. 2015; Wilhelm and Smith 2018; Bergius and Buseth 2019). Foraging species and spaces may be used in restoration and greening, increasing ecosystem functionality (Penone et al. 2012; Bonthoux et al. 2014; Winter and Lucas 2017; Fischer and Kowarik 2020), particularly in areas where ecosystem services are heavily skewed towards human and industrial needs (Botzat et al. 2016; Artmann et al. 2019; Hansen et al. 2019). Multifunctional infrastructure can provide foraging opportunities (Rupprecht et al. 2015; Säumel et al. 2016) and refuge for urban wildlife (Bunge et al. 2019; Menon et al. 2019), enabling responsible production and consumption, climate action, and ecosystem integrity. 

## Conclusion

The study of human foraging behaviour is a relatively nascent field in the context of ethology. Foraging could span the range from a primary lifestyle to an occasional activity, but it confers incremental benefits to those undertaking it. While it does not tie into global economies of scale, it can provide people with important local and contingency alternatives. Foraging relates to local land tenure and knowledge systems that must be respected and reciprocated when promoting this activity. Species and spaces related to foraging can help diversify current agricultural, food, and natural resource systems to make them more adaptive and resilient to short-term shocks and long-term changes.

Concerning sustainable development goals, foraging as a livelihood can reduce poverty and hunger (SDGs 1 and 2) by contributing to food and nutrition security. It can improve human health and wellbeing (SDG 3) through the nature of fresh, nutritious foods and socio-culturally relevant experiences. Foraging can provide economic opportunities and labour dignity (SDG 8) especially to marginalised and vulnerable people, reducing inequalities (SDG 10). Foraging embodies the principles of responsible production and consumption (SDG 12). Designing for and promoting foraging can foster sustainable communities and cities (SDG 11). The locally adaptive nature of foraging systems will enhance efforts towards climate change adaptation, biodiversity conservation, and good governance (SDGs 13, 15, 17).

Promoting foraging as part of a sustainable and healthy lifestyle will require improving equitable access to avenues for foraging, in the form of knowledge and resource systems. Enabling foraging as a sustainable livelihood entails recognising the plurality of personal, economic, social, and ecological values associated with the activity of foraging, and also the resource systems within which it is undertaken. Facilitating landscape management through foraging necessitates exchange of knowledge, praxis, and governance for synergistic outcomes across the sectors of agriculture and food systems, ecological and built infrastructure, and landscape conservation and restoration.

Areas for research include the limits to the complexity of devolved economies and diverse ecosystems in which foraging occurs, non-economic and synergistic values between foraging and ecological conservation and restoration, forms and feasibility of common-pool resource governance, and robustness and adaptive capacity of foraging social–ecological systems. Because access and knowledge are crucial to utilisation, developing foraging infrastructures such as greenspaces and knowledge exchange fora is a priority implementation action. These infrastructures can provide avenues for experimentation and participatory learning for research and resilience.

## Supplementary Information

Below is the link to the electronic supplementary material.

Supplementary file1 (XLSX 3138 KB)

Supplementary file2 (XLSX 1524 KB)
